# Reinfections and Cross-Protection in the 1918/19 Influenza Pandemic: Revisiting a Survey Among Male and Female Factory Workers

**DOI:** 10.3389/ijph.2023.1605777

**Published:** 2023-04-26

**Authors:** Katarina L. Matthes, Mathilde Le Vu, Urmila Bhattacharyya, Antonia Galliker, Maryam Kordi, Joël Floris, Kaspar Staub

**Affiliations:** ^1^ Institute of Evolutionary Medicine, University of Zurich, Zürich, Switzerland; ^2^ Swiss School of Public Health (SSPH+), Zurich, Switzerland

**Keywords:** immunity, Spanish flu, multi-wave pandemic, sex differences, health history

## Abstract

**Objectives:** The COVID-19 pandemic highlights questions regarding reinfections and immunity resulting from vaccination and/or previous illness. Studies addressing related questions for historical pandemics are limited.

**Methods:** We revisit an unnoticed archival source on the 1918/19 influenza pandemic. We analysed individual responses to a medical survey completed by an entire factory workforce in Western Switzerland in 1919.

**Results:** Among the total of *n* = 820 factory workers, 50.2% reported influenza-related illness during the pandemic, the majority of whom reported severe illness. Among male workers 47.4% reported an illness vs. 58.5% of female workers, although this might be explained by varied age distribution for each sex (median age was 31 years old for men, vs. 22 years old for females). Among those who reported illness, 15.3% reported reinfections. Reinfection rates increased across the three pandemic waves. The majority of subsequent infections were reported to be as severe as the first infection, if not more. Illness during the first wave, in the summer of 1918, was associated with a 35.9% (95%CI, 15.7–51.1) protective effect against reinfections during later waves.

**Conclusion:** Our study draws attention to a forgotten constant between multi-wave pandemics triggered by respiratory viruses: Reinfection and cross-protection have been and continue to be a key topic for health authorities and physicians in pandemics, becoming increasingly important as the number of waves increases.

## Introduction

Currently in the third year of the COVID-19 pandemic caused by the SARS-CoV-2 virus, questions about reinfection and immunity resulting from vaccination and/or previous illness in the light of a mutating virus are of great relevance (1). Similar questions that have arisen regarding earlier pandemics, however, have only received marginal attention. The 1918/19 influenza pandemic (A/H1N1) has already elicited a small number of investigations about reinfections and immunity (2, 3). Using a previously unnoticed archival data source from the 1918/19 pandemic among factory workers in French-speaking Switzerland, we would like to draw attention to similar, comparative discussions addressing cross-protection and reinfection between past, present and future pandemics.

A review of the existing literature shows that only selective information is available on whether the virus mutated over time in 1918/19 and possible multi-wave reinfections. There is increasing evidence from lung specimens that the viral sequence changed throughout the pandemic (4): two amino acid differences on the influenza nucleoprotein were found when comparing pre-pandemic and pandemic peak European strains from 1918, suggesting that 1918 influenza viruses adapted their ability to escape the host`s innate immune response. In historical archival sources, few studies investigated reinfection and cross-protection between waves in 1918, but all of them suggest that reinfections did occur in a low percentage range. One study analysed data from 16,125 English civilians and found that 1.7% reported multiple infections (2). Another study reported similar figures among British civilians when 1% of people reported a reinfection, whereas <1% of the 90,000 sailors of the British Grand Fleet appeared ill in both summer and fall waves (5). However, the underlying data sources and methods of these studies differ considerably.

Most of the historical-epidemiological studies mentioned above are statistical and survey-based studies conducted by health authorities or physicians. Since the 1889/90 pandemic, it has been standard practice to obtain health-related data from physicians following pandemics via surveys (6–8). However, the start of survey distribution and data collection among the general population at the beginning of the 20th century contributed to the larger picture of emerging disease statistics, particularly with regards to morbidity data. Switzerland was hit by the 1918/19 pandemic in several waves (9–12). Over the course of the Spanish Influenza pandemic, the disease claimed approximately 25,000 lives in Switzerland; as confirmed by recent estimates of excess mortality (12, 13). Contemporary studies estimate that, including unreported cases, around 2/3 of the Swiss population fell ill during this time (14).

Factories have been identified by health authorities as key transmission sites since the 1890s. Pandemics usually affect those of lower socioeconomic strata more severely, and the literature shows that this was also the case in 1918/19 (7, 15). Whether this social gradient also applies to reinfections is unclear. In this study, we analysed a health survey among the entire workforce of a large factory in Cossonay, Switzerland, a relatively homogeneous population from a lower socioeconomic status with close spatial proximity. We investigate the incidence of influenza-like-illness (ILI, used in this article without precise definition as a collective term for flu-like illnesses) among factory workers during the 1918/19 pandemic according to sex and age, incidence of reinfections, illness and reinfection severity, and whether illnesses in the first waves and during the 1890 pandemic had a protective effect during the subsequent waves in 1918.

## Methods

The district of Cossonay (12,020 inhabitants in 1920) is located in the hinterland of Lake Geneva and the French-speaking canton of Vaud (16). The district had 15 factories in the 1920s, but in terms of worker number, the Aubert and Grenier cable factory (founded in 1898) was by far the largest in the district (17). In 1918, approximately 820 people worked in this factory, of which about 70% were males workers, 25% were female workers, and 5% were tradesmen and technicians (18–20). Compared with the other Swiss cantons, the canton of Vaud was moderately affected by the 1918/1919 pandemic. Among the 317,457 inhabitants of Vaud, 2,221 (0.70%) died of influenza (14, 21). In 1919, following the pandemic, the Vaud authorities estimated that around 55% (*n* = 175,000) of the population were infected during this time in the canton (22). Further information regarding the course of the Pandemic in Cossonay, implemented interventions, and information on historical time context can be found in the Supplementary Material.

Dr. Alfred Renaud was one of two physicians in Cossonay district and the district’s officially delegated medical doctor. In the summer of 1919 (exact date unknown), he conducted a survey of all the workers and employees in the Aubert Grenier cable factory. The few historical sources that have survived make it seem as if the survey was initiated by Alfred Renaud himself, rather than being commissioned by the factory or the authorities (23). However, the motivation for the survey remains unclear. In the autumn of 1919, Alfred Renaud wrote a letter to the cantonal authorities to ask whether he could bill them for the analysis of the survey in his role as delegated medical doctor, which was confirmed by the canton. In the spring of 1920 he then sent the authorities a four-page handwritten letter containing more information about the survey (we consider this letter in more details in the discussion) and a descriptive analysis (23).

This letter shows that the survey was carried out among all 820 workers and employees of the factory in the summer of 1919, representing the entire factory population. The questionnaires were filled out during a nurse’s visit at the factory (the different handwriting on the individual questionnaires suggests that the employees filled out the forms themselves). The exact interview process and the additional information that the nurse might have given to the employees is not known. The 820 questionnaires and the two letters mentioned above have been preserved in the Vaud State Archives (Archives cantonales vaudoises ACV, signature KV III b 27/1 and 27/2) (23, 24). There was a differently coloured questionnaire for each sex (an example is reproduced in Supplementary Figure S4). Age was given in years (continuous, but we also categorized it as under versus over 40 years). Then it was asked whether the person had the flu (yes/no), whether they had the flu more than once (yes/no), whether the illnesses were mild or severe, and when the person fell ill (summer wave in July/August 1918, autumn/winter wave October-December 1918, or early 1919). We have combined the two categories October/November 1918 as well as December 1918 into one wave/category. At the end, the question was asked exclusively for those over 30 years of age, whether they had also been infected by the flu in 1889/90. A letter sent to cantonal authorities by Dr. Renaud indicates that three factory personnel died of influenza during the pandemic, accounting for approximately 0.7% of those who were infected, or around 0.4% of the entire factory workforce (23).

### Statistical Methods

First, we described the data in terms of number of infections, age distribution, reinfections and illness severity. All factors were also analyzed separately by sex. Secondly, we estimated the effect of age groups and sex on illness in general, specifically illness in the first wave (summer 1918) and second wave (fall/winter 1918 and early 1919); and on the severity of the illness using a logistic regression model. The latter model was additionally adjusted for age, and the results are presented as Odds Ratio (OR) with 95% confidence intervals (CI). Thirdly, we calculated the cross-protection of A) illness in wave 1 (July and August 1918) against illness in wave 2 and 3 (October to December 1918 and in early 1919) and B) illness in 1890 against illness in 1918/19. The relative risk (RR) and the associated 95% CI was estimated. The protection effect was calculated as 1-RR.

## Results

The study population consists of all 820 workers at the Aubert and Grenier cable factory of which 212 were female (25.9%) and 608 were male (Table 1). Overall, 25.6% of the workers were 40 years old or older, while 74.4% were younger than 40. The age distribution between men and women differs significantly: female employees were on average younger (26.3 years vs. 33.3 years, *p* < 0.001). 50.2% (*n* = 412, 95% CI 46.8–53.7) of all employees reported illness during the 1918/19 pandemic. Among male workers 47.4% reported an illness vs. 58.5% of female workers, leading to a significantly higher proportion of illness in females compared to males (difference of 11.1 percent points, 95% CI 3.01 to 19.2, *p* < 0.001). Overall, 84.0% of workers reporting an illness were under 40 years old. In terms of reported severity, 57.8% of the 412 reported illnesses were categorized as severe and 41.3% as mild with no significant difference between the sexes (*p* = 0.925). The majority of infections (91.8%) occurred either in the first or second waves, with similar numbers of infections reported in both waves, the rest of the illnesses occurred in the first months of 1919. The proportion of infections among those over 40 years of age decreased slightly in each subsequent wave.

**TABLE 1:** Descriptive statistics of the survey (Cossonay, Switzerland. 1919).

	Men		Women		Total	
	*n*	%	*n*	%	*n*	%
A) Sample size (persons)	608		212		820	
Age						
Mean age (y) (sd)	33.3 (12.7)		26.3 (10.1)		31.5 (12.4)	
Median age	31		22		28.5	
Range (y)	15 to 88		15 to 77		15 to 88	
Aged <40 years	422	69.4	187	88.2	610	74.4
Aged ≥40 years	186	30.6	25	11.8	210	25.6
Persons reporting flu illnesses						
Total	288	47.4	124	58.5	412	50.2
Thereof aged <40 years	231	80.2	115	92.7	346	84
Thereof aged ≥40 years	57	19.8	9	7.3	66	16
Sickness rate (%) in persons aged <40 years		54.7		61.5		56.8
Sickness rate (%) in persons aged ≥40 years		30.6		36.0		31.3
Flu illness severity						
Persons reporting severe illness (es)	166	57.6	72	58.1	238	57.8
Persons reporting mild illness (es)	119	41.3	51	41.1	170	41.3
Unclear	3	1	1	0.8	4	1
Total	288	100	124	100	412	100
Ill persons reporting reinfection(s)						
Total	44	15.3	19	15.3	63	15.3
Thereof aged <40 years	32	72.7	19	100	51	81
Thereof aged ≥40 years	12	27.3	0	0	12	19
Reinfection rate (%) in ill persons aged < 40 y		13.9		16.5		14.7
Reinfection rate (%) in ill persons aged ≥ 40 y		21.2		0.0		18.2
Reinfection severity						
2nd illness was equally strong/stronger	36	81.8	14	73.7	50	79.4
2nd illness was weaker	6	13.6	2	10.5	8	12.7
Unclear	2	4.6	3	15.8	5	7.9
Persons reporting flu illness in 1890	11	1.8	2	0.9	13	1.6
B) Flu illnesses reported (incl. reinfections)						
Total flu illnesses	338	100	146	100	484	100
Wave 1 (July/August 1918)						
Total flu illnesses Wave 1	151	44.7	69	47.3	220	45.5
Thereof aged ≥40 years	38	25.2	5	7.2	43	19.5
Thereof reinfections	8	5.3	5	7.2	13	5.9
Wave 2 (October-December 1918)						
Total flu illnesses Wave 2	159	47	65	44.5	224	46.3
Thereof aged ≥40 years	25	15.7	4	6.2	29	12.9
Thereof reinfections	34	21.4	15	23	49	21.9
Early 1919						
Total flu illnesses early 1919	17	5	5	3.4	22	4.6
Thereof aged ≥40 years	3	17.6	0	0	3	13.6
Thereof reinfections	6	35.3	1	20	7	31.8
Unclear	11	3.3	7	4.8	18	3.7

The upper part (A) shows the data by persons who completed the questionnaire. The lower part (B) resolves the data by flu illnesses (the total number of flu illnesses is higher than the number of persons due to reinfection).

**FIGURE 1:**
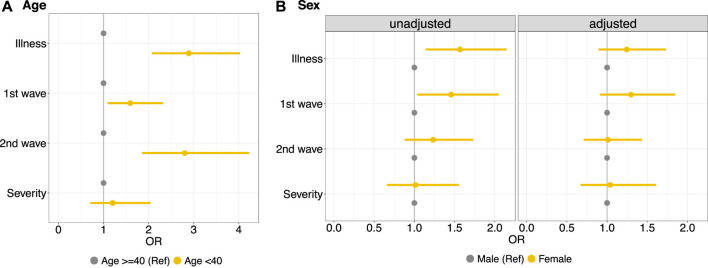
Left side, **(A)** Logistic regression model to estimate the effect of age groups (<40 years vs. ≥40 years) on illness in general, specifically illness in the first wave (summer 1918) and subsequent waves (fall/winter 1918 and early 1919); and on the severity of the illness. Right side, **(B)** Logistic regression model to estimate the effect of sex on the same outcomes, unadjusted (left panel) and adjusted for age (right panel) (Cossonay, Switzerland. 1919). OR, odds ratios.

The results of the logistical models illustrate that workers aged <40 years had, in general, a higher risk of getting ill than those of 40 years of age or older (OR 2.89, 95% CI 2.07–4.03), as suggested by the observed greater risk of illness in the first two waves. There is no evidence of higher risk of severe illness for workers <40 years of age compared to workers ≥40 years (Figure 1A). Considering the unadjusted results for age, it is evident that female workers have a greater risk of illness (OR 1.56, 95% CI 1.14–2.15) compared to male workers (Figure 1B, unadjusted). Age-adjusted results, however, did not show any evidence of increased infection risk for female workers (OR 1.24, 95%CI 0.89–1.73) (Figure 1B-adjusted). This is due to the fact that female workers in this factory were significantly younger than male workers (Supplementary Figure S5) and therefore the risk of illness in the factory was rather affected by age than by sex.

Among those who reported illness (*n* = 412), 15.3% (*n* = 63, 95% CI 12.0–19.2) reported a reinfection and thus several illnesses between July 1918 and the beginning of 1919. This proportion was exactly the same for men and women. While 54 people reported two infections, 9 people reported being sick even three times. In the majority of the cases (79.4%), the subsequent infection was reported to be as strong as, or stronger than the first illness. Over the three waves, the proportion of reinfections increased steadily from 5.9% in the first wave to 21.9% in the second wave, to 31.8% in early 1919. Illness during the first wave in July and August 1918 was associated with 35.9% (95% CI 15.7–51.1) protection against illness in the second wave (October to December 1918) or in early 1919, relative to those who were not ill in wave 1 (Table 2).

**TABLE 2:** Cross-protection of illness for A) wave 1, 1918 against illness in wave 2 and 1919 and B) cross-protection of illness in 1890 against illness in 1918/19 for those ≥30 years (Cossonay, Switzerland. 1919).

A) Illness in wave 1 (July/August 1918) vs. illness in subsequent waves 2 (October-December 1918) or 1919			
	Wave 2 or 1919 illness	Wave 2 or 1919 healthy	Total
Wave 1 illness	45	175	220
Wave 1 healthy	191	408	599
Total	236	583	819

RR = 0.641 (95%CI 0.489–0.842); Protection = 35.9% (95%CI 15.7–51.1).

**Table:** 

B) Illness in 1890 vs. illness in 1918 or 1919 (only among those ≥30 years)			
	1918/1919 illness	1918/1919 healthy	Total
1890 illness	3	10	13
1890 healthy	158	217	375
Total	161	227	388

RR = 0.548 (95%CI 0.212–1.41); Protection = 45.2% (95%CI −41.6–78.8).

Only 13 (3.4%) of the *n* = 388 workers over 30 years of age reported being ill during the 1890 pandemic (11 men and 2 women). Among them, 3 also reported being ill during the 1918 pandemic (Table 2). Thus, illness during the 1890 pandemic was associated with 45.2% (95% CI −41.6%–78.8%) protection against illness during the 1918/1919 pandemic relative to those of the same age range who did not report illness in 1890. However, due to the small number of recalled cases for illness in 1890 (see the width of the confidence interval), this result should not be overemphasized.

## Discussion

We revisited individual responses to a survey on the 1918/19 pandemic among factory workers in Western Switzerland, conducted by a physician in mid-1919. Slightly more than half of the factory workforce reported having had the flu at least once between July 1918 and early 1919. The majority of illnesses were perceived as severe, and workers under 40 years of age were more affected. Women were proportionately more affected than men, although this can be explained by a difference in age distribution: female workers were significantly younger than male workers, suggesting that the difference in infection risk was more driven by age than by sex. About one in eight factory workers fell ill more than once, and four out of five reinfections were more severe than the first infection. We additionally find evidence of cross-protection: illness in the first wave, in summer 1918, was associated with protection against illness in the subsequent waves.

Our study expands the knowledge from the 1918/19 pandemic in Switzerland. During the 1890 pandemic, and again in 1918, factories were explicitly perceived as transmission hotspots in reports by the federal and cantonal authorities (22, 25). As a result, factory managers were instructed to keep records of cases of illness and also to report them to the authorities, in accordance with the obligation to report influenza cases (25). However, with regard to the comparison of infection rates with the general population, it should be noted that only few interventions were implemented in factories at that time (very few closures, as compared with the gathering bans and school closures among the general population) (26). The fact that more than 50% of the factory population fell ill fits well with the limited information on factories in Switzerland at that time: In a large factory in the canton of Valais, consisting of about 2000 workers, 20% fell reportedly ill, while in two medium-sized factories with approximately 300 workers each, 50% and 70% fell ill, respectively (27, 28). This range of infection rate in factories corresponds to the estimated infection rates by authorities for the entire canton of Vaud (55%) as well as for the general population of Switzerland (66%) (14, 22). Additionally, infection rate estimates for army troops in Switzerland during the summer wave of July/August 1918 are similar, with an infection rate of 53% (29, 30).

In the factory population, the reinfection rate was approximately 15% in 1918/19, which is higher than the few comparative figures in the low percentage area offered by the literature (2, 5). The lower magnitude of reinfections from the literature would actually correspond better to the qualitative information offered by a large survey among physicians conducted by the canton of Vaud in 1919 (22). Approximately half of the 118 surveyed physicians that observed reinfections, stated that these were rare occurrences. On the one hand, a direct comparison with the reinfection rates from the literature is challenging because of differences in data sources and methods (underreporting could also play a role). On the other hand, the self-reported reinfections in Cossonay could also include relapses as well as overreporting. However, it seems that the reinfection rate in this particular factory subpopulation of close spatial proximity was rather high. Studies have shown that lower socio-economic groups are disadvantaged in past and present pandemics in terms of morbidity and mortality, partly due to working conditions (7, 15). This could also apply to reinfection rates, as towards the end of the First World War, the nutritional status of the Swiss population and especially in the middle and lower socio-economic strata began to suffer increasingly, people became measurably thinner, which probably also affected their resistance and the building up of immunity after illness (31).

In both British civilian communities and troops, having had the flu during the first 1918 pandemic wave provided a 35%–72% protection against illness in the fall wave (5). The 36% protective effect among factory workers in this study is thus in the lower reported range of protection in the literature. For example, based on hospital admissions due to pneumonia and/or influenza among soldiers of the Canadian Expeditionary Force during the fall wave, it was shown that being a seasoned soldier provided an 83% protective effect from developing ILI, and an 84% protective effect against ILI-related mortality (32). Since only three workers died from the flu among the factory population, the amount of cross-protection conferred by an infection during the summer of 1918 against mortality in subsequent waves cannot be calculated. In these cases, the time of death and rates of reinfection were also unknown. It is worth noting that the three fatalities among the Cossonay factory workers correspond to a case-fatality rate (CFR) of 0.7%, which is lower than the CFR usually reported for the 1918 pandemic, however greater than the CFR of other influenza pandemics and seasonal waves (33).

Homologous reinfection is a possible driver of multiple-wave influenza outbreaks, as has been suggested for the 1971 influenza epidemic within the isolated population in Tristan da Cunha (34): A pattern of reinfection was observed during the two-wave outbreak, suggestively attributed to host heterogeneity in immune response, where the delay or absence in a humoral response in certain individuals resulted in reinfection by the same influenza strain or a related variant. This delay in the development of long-term immunity or weak immune response can be explained by the varying levels of antibody production: Those who develop fewer antibodies are more susceptible to reinfection by similar variants and thus fail to achieve an effective antibody level to provide long-term immunity, which could play an additional role especially in subgroups with close spatial proximity (isolated populations, schools, factories, etc.)

In our study, we find a higher proportion of illness in younger factory workers <40 years old, as compared to factory workers ≥40 years of age. Although there is large evidence in the literature that young adults suffered the highest death rate during the 1918/19 pandemic, few studies focused on morbidity and incidence disaggregated by age. Therefore, the comparison with the literature is rather difficult (35). However, this age pattern in infection rates could be explained by the fact that young people either had no contact with a pandemic influenza virus in the years before the H1N1 1918/19 pandemic, or that they had only been in contact with the “Russian flu” of 1889/90, which is supposed to have been caused by an H3 influenza virus. The exposure to this H3 virus would then not have conferred immunity or protection from later exposures to different flu subtypes, including the H1N1 1918/1919 pandemic (36, 37). A similar pattern was observed in the A/H1N1 influenza pandemic in 2009, when younger people also had a higher rate of infection (38–43), and they also had little contact with a pandemic influenza virus in the preceding decades. The extent to which these age patterns can be applied to other 20th century pandemics (44, 45) would require more in-depth literature review, as age-specific immunity across pandemics and subtypes of the influenza virus tends to be complex (46, 47).

A direct comparison between the 1918/19 influenza pandemic and the COVID-19 pandemic triggered by the SARS-CoV-2 virus is difficult among other things, because the viruses are different, the time context is shifted by more than 100 years, and a widespread use of vaccines has been influencing events since the end of 2020 (48, 49). While the influence of viral mutations on current pandemic events has proven to be great today, knowledge on the 1918/19 pandemic is still limited on this question (4). Nevertheless, the approximately 15% estimated reinfection rate is similar to the estimated rate of SARS-CoV 2 reinfections during the current COVID-19 pandemic (50, 51). Moreover, the increase in reinfection rate from wave to wave is described, at the moment, for SARS-CoV-2, and we show it here for a 1918/19 factory population as well. The question of whether older people were less affected than younger age groups during the 1918/19 pandemic (because they had experienced the “Russian flu” 1889/90) was discussed by contemporary authorities and physicians in 1918 (22). Our evidence from the factory is thin on the ground due to small sample size. Until the causative pandemic virus is identified in an archival collection specimen, it must even remain an open question whether the “Russian flu” was caused by an influenza virus at all (52, 53).

Our study has several limitations. Firstly, it is based on a relatively small sample of employees from a single factory in Western Switzerland. The fact that this sub-population is more likely to represent the lowest socio-economic stratum causes difficulties regarding generalizability of the results. Although the questionnaires were completed by factory workers during a nurse visit, they are nevertheless based on self-reporting, as are the subjective assessments of the illness severity. Needless to say, reported illnesses are not laboratory-confirmed and can only be considered influenza-like-illnesses. Thus, there is also the possibility of under- and over-reporting of illness. Also, it should be mentioned again that we could not apply any of the definitions of ILI that are in use today to these historical data, since the historical sources do not mention any symptoms or a definition of their own. Furthermore, the understanding of flu at that time may well differ from today’s understanding. Additionally, among reported reinfections during the first wave in summer 1918, there was also the possibility of resurgences. Unfortunately, we do not have detailed information on the exact timing of the illnesses (duration, days of absence, etc.), thus, the influence of the pandemic on factory operations *via* sick days or lengthy absences (post viral syndrome) cannot be assessed. Even though we know that the factory was never closed during the 1918/19 pandemic, other possible protective measures implemented by the factory management are unknown.

Our study suggests that reinfection played an increasingly large role from wave to wave in 1918/19, perhaps even more so in socially disadvantaged groups of close proximity than in the general population. Additionally, the results indicate that infection during a first wave nevertheless offered some protection against infections in subsequent waves. The past holds scenarios that must be taken into consideration and integrated into solutions for current and emerging challenges. Our study points to a forgotten constant between multi-wave pandemics triggered by respiratory viruses: Reinfection and cross-protection have been and continue to be a topic for authorities and physicians in such settings, becoming increasingly important as the number of waves and the time between these increase.

## Data Availability

The datasets presented in this study and the codes can be found in online repositories: Github: https://github.com/KaMatthes/Reinfections_and_cross_protection_1918_influenza Zenodo: https://doi.org/10.5281/zenodo.7274470.
