# Supplementary material for: Reinfections and Cross-Protection in the 1918/19 Influenza Pandemic: Revisiting a Survey Among Male and Female Factory Workers
Source: Int J Public Health. 2023 Apr 26;68:1605777. doi: 10.3389/ijph.2023.1605777 (PMC10169597; doi:10.3389/ijph.2023.1605777)
Supplement: Supplementary file 1 [file Presentation1.pdf]

SUPPLEMENTARY MATERIAL

Reinfections and cross-protection in the 1918/19 influenza pandemic: Revisiting a survey among male and female factory workers

---

Index

Supplementary information on data and methods 2
Supplementary Figures 4
Supplementary Tables 9
Bibliography 11

Supplementary information on data and methods

Cossonay and the Aubert Grenier cable factory around 1918

The district of Cossonay had 12,020 inhabitants in 1920 according to the census. It is located in the hinterland of Lake Geneva and the French-speaking canton of Vaud, and consisted of 33 municipalities in 1920 (Map Supplement Figure S1) (Schweiz. Statistisches Bureau). One of these communities is Cossonay itself, with the district principal place Cossonay (approximately 1’200 inhabitants). The district of Cossonay had 15 factories in the 1920s, but in terms of workers number, the Aubert Grenier cable factory was by far the largest in the district (Eidgenössisches Statistisches Amt 1930). This factory was founded in 1898 and mainly produced cables; its buildings were located near Cossonay railway station. In 1918, about 820 people worked in this factory, of which about 70% were males workers, 25% were female workers, and 5% were tradesmen and technicians (1928, 1948; Imsand 1972). It can be assumed that the vast majority of workers and employees lived in the immediate vicinity of the factory. Photographic impressions of the factory from around 1918 can be found in Supplementary Figure S2 (1928, 1948; Imsand 1972).

The 1918/19 pandemic in the Canton of Vaud and Cossonay

Around 1920, Switzerland consisted of 25 federal member states, the so-called cantons. Compared with the other Swiss cantons, the canton of Vaud was moderately affected by the pandemic of 1918/1919. Among the 317,457 inhabitants, 2,221 people died of influenza (0.70%), 49.4% of whom were men and 31.7% (and thus over-proportionally many) were between 20 and 40 years of age (Eidgenössisches Statistisches Bureau 1919; Sonderegger 1991). The course of the pandemic in the canton of Vaud followed the so-called “western Swiss pattern” as defined by Sonderegger 1991 (Sonderegger 1991), i.e. an early and relatively strong summer wave, followed by a long and, again, strong autumn/winter wave. After the pandemic in 1919, the Vaud authorities estimated that around 175,000 cases of influenza had occurred in the canton (Autorités de la santé du canton de Vaud 1919), implying that around 55% of the population had fallen ill.

Compared to other Swiss cantons, the canton of Vaud introduced the compulsory reporting of influenza relatively late, on September 24th 1918, i.e. after the summer wave (Messerli 1920; Koch 2019). Therefore, weekly reports of new cases can only be used as an indicator for the course of the pandemic from then on. In the district of Cossonay, 2,656 cases and 35 deaths from influenza were reported between the end of September 1918 and the end of March 1919 (around 800 more cases occurred in the summer wave before September 1918) (Supplementary Table S2 and Supplementary Figure S3) (Schweiz Schweizerisches Gesundheitsamt). For the period following September 1918, the incidence curves in the entire canton and in the district of Cossonay coincide relatively well. Only the resurgence after the large national workers' strike in November 1918 was stronger in Cossonay than in the canton as a whole. In order to depict the summer wave, weekly death figures from the city of Lausanne and hospitalisations in the entire canton must be used as a proxy.

In Switzerland, cantons were responsible for interventions throughout the pandemic (Staub et al. 2021). The non-pharmaceutical interventions (NPI) taken by the canton of Vaud are shown in Supplementary Table S1. In the summer wave in July/August 1918, schools were sent on summer holiday earlier (week 28), followed by a ban on gatherings (week 29), and health workers being advised to wear masks around infected patients (week 31). In September 1918, relaxation between waves followed when, in week 36, the ban on gatherings was lifted. At the end of September 1918, the reporting obligation for influenza was introduced.

When the second and strong wave picked up in early October 1918, the authorities again reacted with school closures and limited hours of operation for restaurants and other public places (week 41). Only one week later (week 42), the measures had to be tightened and a new ban on gatherings was imposed. At the beginning of November, as everywhere else in Switzerland, there were army troop gatherings and mass gatherings of strikers in Lausanne and the canton of Vaud as part of the national socio-political and labour strike, which led to a

renewed resurgence of the epidemic. Afterwards, the long and violent autumn wave slowly subsided and by mid-November 1918 the communes were able to reopen their schools (in Cossonay the schools did not reopen until early December). In Switzerland, in the autumn/winter of 1918, most cantons decided that factory owners and managers had to report cases of illness among the workforce to the authorities and ensure that sick workers stayed away from work long enough until they were well again. However, for economic reasons, the cantons explicitly refrained from closing factories and did so only in individual cases .

After the pandemic in mid-1919, the health authorities of the canton of Vaud carried out a review of the pandemic (Autorités de la santé du canton de Vaud 1919). This was done within the framework of a detailed questionnaire survey to all physicians in the canton. 118 physicians responded to the 15 questions, some of which were detailed, about the course of the pandemic and the disease. These questionnaires were processed into a report. In this report, factories were also identified as important transmission sites in addition to schools and other locations (it was written that influenza spread with rapidity among the working population, who returned to their families, contaminating them and eventually the localities around them). A subchapter also explores the question of how the 1890 and 1918 pandemics might be related. It is assumed that due to the relatively less affected older population groups in 1918, there was immunity provided by infection during the 1890 pandemic among the older population in 1918.

Another question to physicians was whether they observed reinfections and relapses *(«Avez-vous observé des récidives? Faire la distinction entre récidive et rechute.»)*. Among the 118 questionnaires sent back, 56 physicians had observed reinfections, saying that these were rather rare. Fifty doctors had not observed reinfections, and 12 did not answer the question. Dr. Alfred Renaud, the delegated physician in the district of Cossonay, had also observed several reinfections. For example, the case of a young woman, getting sick in July 1918, then in perfect health for 2.5 months, caring for her sick husband in November 1918, and getting reinfected and developing pneumonia. The conclusion of the sub-chapter on reinfections in the report was that reinfections occur (because, “,the immunisation of a previous illness is not perfect, which is why there are seasonal flu epidemics”), although quite rarely and in the single-digit percentage range, with some of these reinfections being more severe than the first infection.

The 1918/19 pandemic in the Aubert Grenier factory

Unfortunately, no archival materials from the factory survived that would allow conclusions to be drawn about how exactly the factory management reacted to the pandemic in the autumn/winter of 1918. There are no surviving lists of workers' names or statistics on absent days or anything similar. The surviving minutes of the few board meetings in the second half of 1918 and the first half of 1919 always speak of normal factory operations and do not mention the 1918 pandemic or its effects. Although there is talk of supply problems with raw materials towards the end of the First World War, in the archived financial operating results, the year 1918 fared better than prior and subsequent years.

Supplementary Figures

Supplementary Figure S1: The districts of Switzerland ca. 1918, red = the district of Cossonay (Cossonay, Switzerland. 1919).

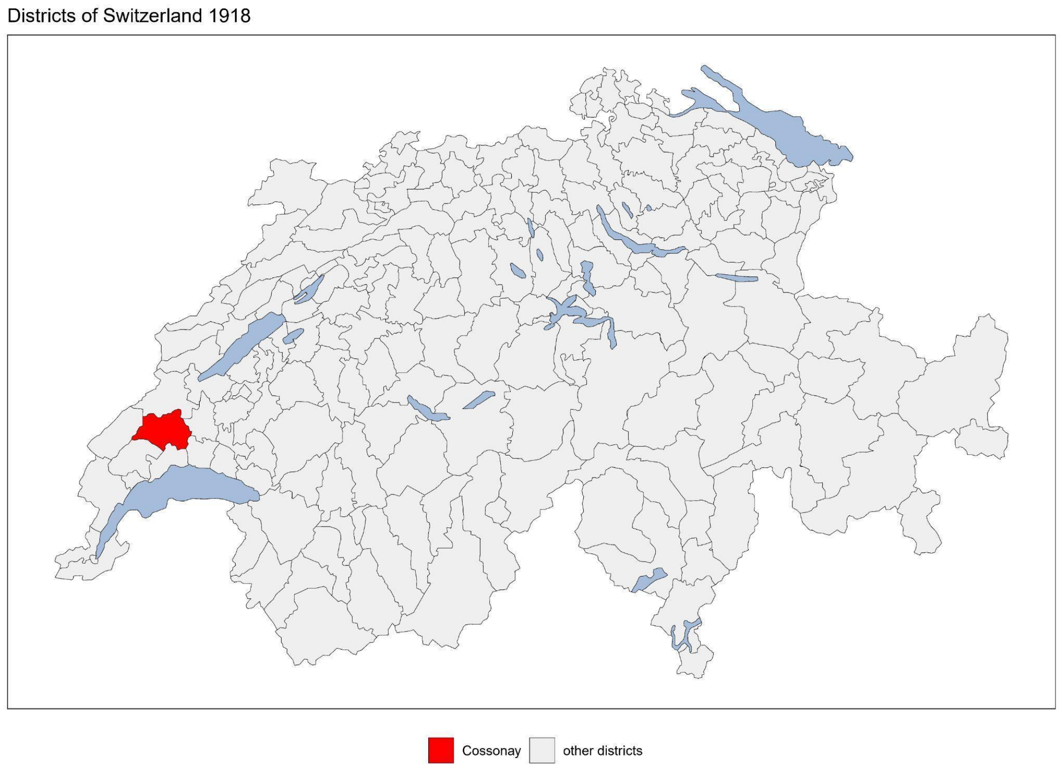

Supplementary Figure S2: Photographs of the Aubert & Grenier factory in Cossonay from the period around the 1918 pandemic (Cossonay, Switzerland. 1919): A) The entrance to the factory, ca. 1910; B) Side view of the factory, ca. 1910; C) A female factory worker at the telephonic wire wrapping machine, early 1920s; D) A male factory worker at the cable twisting machine, ca. 1920; E) The factory canteen, ca. 1920 (Source: Archives cantonales vaudoises, inventory number PP 632, Société anonyme des câbleries et tréfileries de Cossonay).

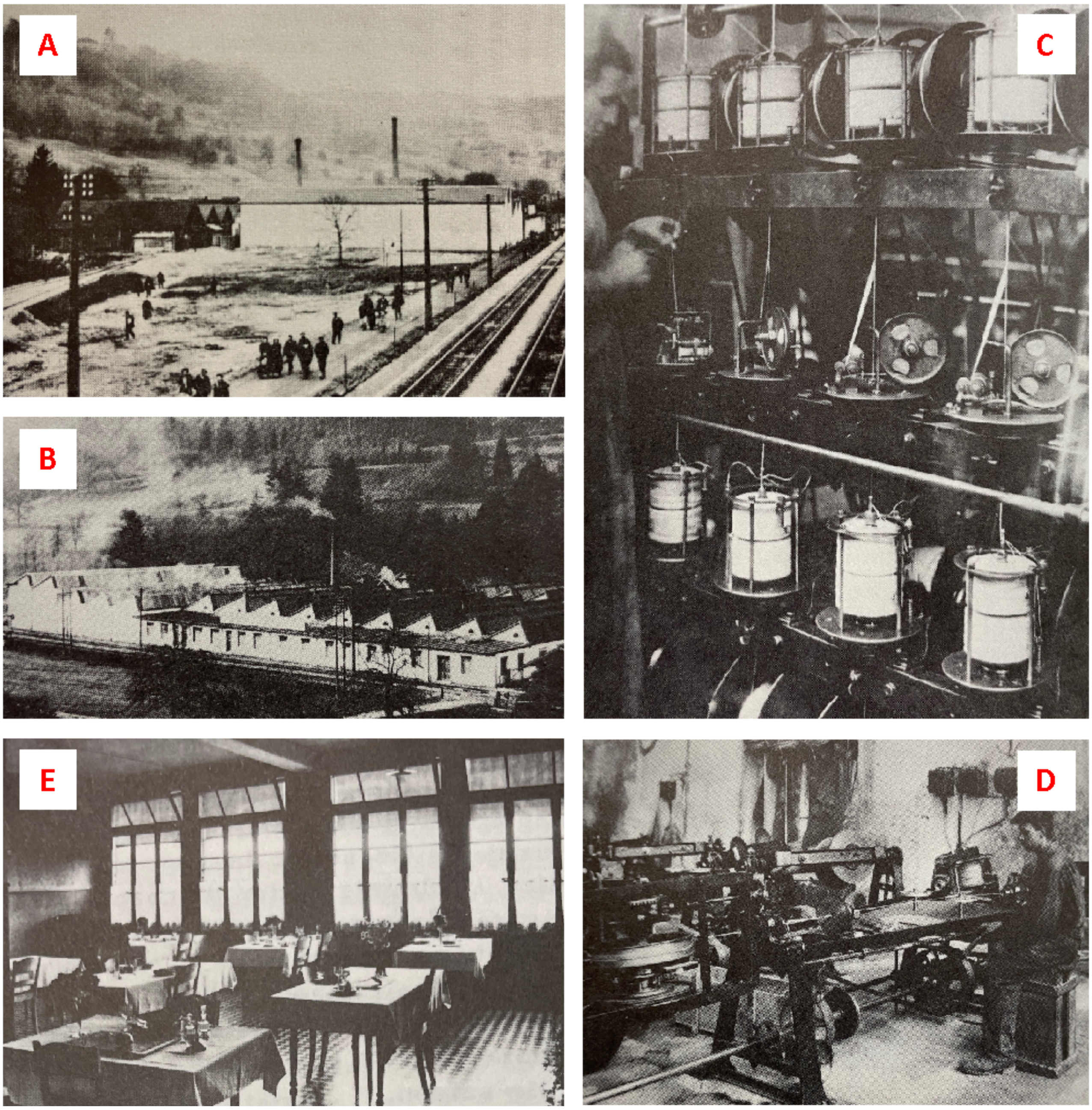

Supplementary Figure S3: Various parameters per calendar week showing the course of the pandemic in the canton of Vaud (Canton of Vaud/Lausanne/Cossonay, Switzerland. 1919): A) Newly reported cases of flu for the whole canton of Vaud and for the district of Cossonay (the dashed line indicates the introduction of the cantonal reporting obligation on 24.09.1918); B) New deaths (all causes) for the city of Lausanne; C) New hospitalisations for the whole canton of Vaud (total and due to the cause "other infectious diseases", which also includes influenza). The solid line at the end of September 1919 indicates the approximate date of the factory survey.

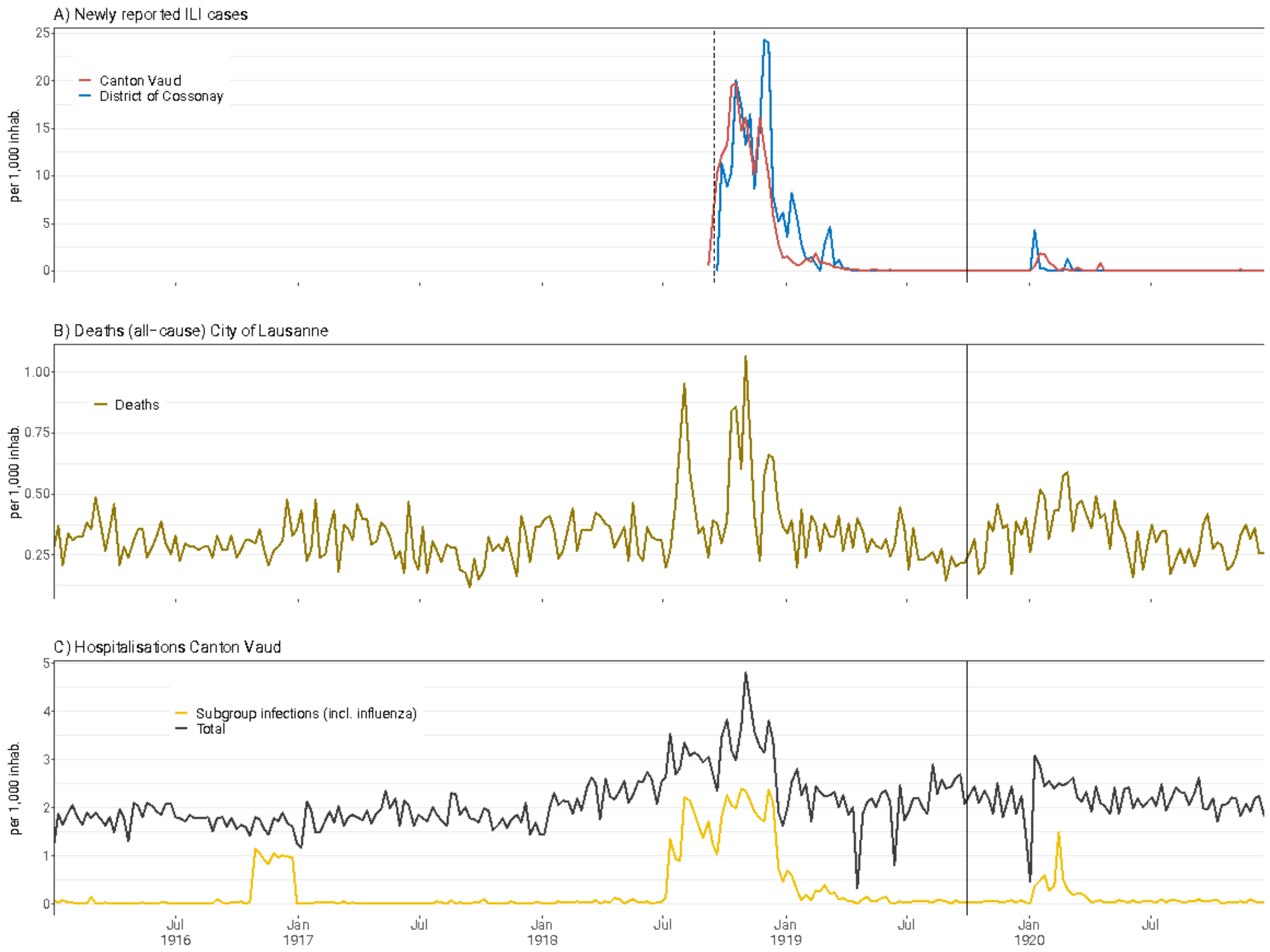

Supplementary Figure S4: An example of a survey sheet for women (those for men were printed on red paper and were otherwise identical) (Cossonay, Switzerland. 1919). Unfortunately, the names or initials or personal numbers at the beginning could not be linked to any other archival source from the factory.

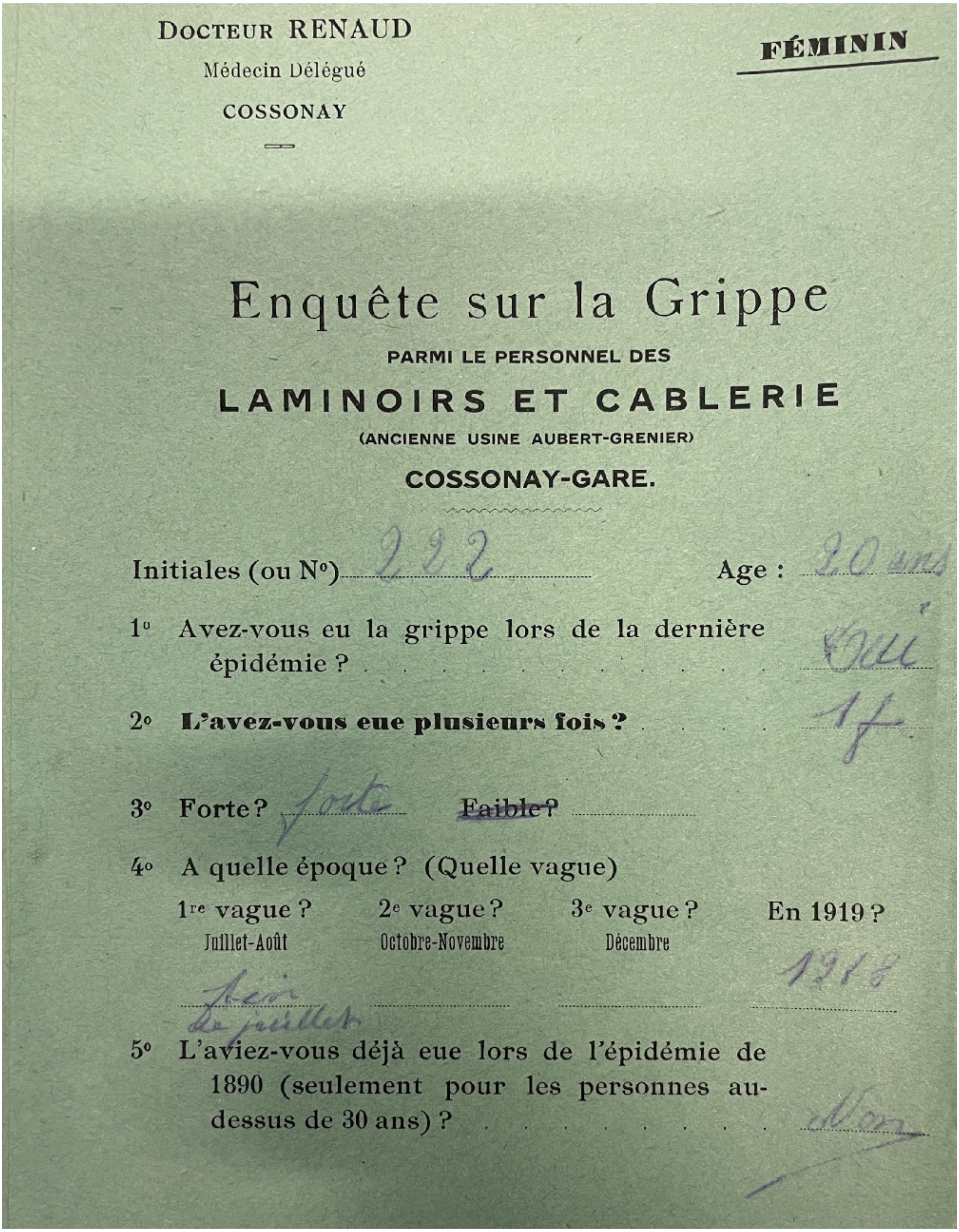

DOCTEUR RENAUD
Médecin Délégué
COSSONAY

FÉMININ

Enquête sur la Grippe
PARMI LE PERSONNEL DES
LAMINOIRS ET CABLERIE
(ANCIENNE USINE AUBERT-GRENIER)
COSSONAY-GARE.

Initiales (ou N°) 222 Age : 20 ans

1° Avez-vous eu la grippe lors de la dernière épidémie ? . . . . Oui

2° **L'avez-vous eue plusieurs fois ?** . . . . . [illegible]

3° Forte ? forte ~~Faible?~~

4° A quelle époque ? (Quelle vague)

1re vague ? Juillet-Août — [illegible] de juillet

2e vague ? Octobre-Novembre

3e vague ? Décembre

En 1919 ? 1918

5° L'aviez-vous déjà eue lors de l'épidémie de 1890 (seulement pour les personnes au-dessus de 30 ans) ? . . . . Non

Supplementary Figure S5: Age distribution among female (n=212) and male (n=608) factory workers in Cossonay, black bar = median age (22 years in female workers, 31 years in male workers) (Cossonay, Switzerland. 1919).

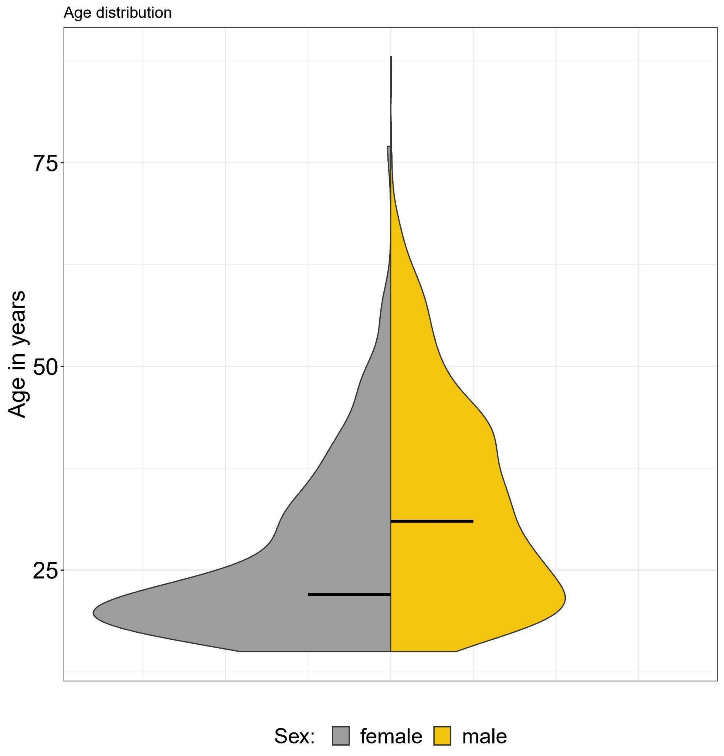

Supplementary Tables

Supplementary Table S1: Non-pharmaceutical measures implemented by the Vaud cantonal authorities (Canton of Vaud, Switzerland. 1919). The Swiss Federal authorities barely did not impose measures.

Date	Calendar week	Interventions at the level of the canton Vaud
10.07.18	28	Schools are sent on summer holiday earlier.
19.07.18	29	Ban on gatherings: Dancing and sporting events, cinema, festivities, competitions, assemblies, etc. are prohibited.
03.08.18	31	Health workers advised to wear masks around infected patients.
06.09.18	36	Relaxation of the ban on gatherings (cinemas etc. allowed again).
24.09.18	39	Introduction of the reporting obligation for influenza.
12.10.18	41	Schools closed again, restaurants and other public places only open until 21:00.
17.10.18	42	Ban on gatherings: Assemblies of any kind are prohibited.
10.11.18	46	National general strike of workers (public gatherings & large deployments of Armed Forced troops all over Switzerland)
11.11.18	46	The municipalities can open the schools again

Supplementary Table S2: Weekly reported new flu cases and deaths from flu in the district of Cossonay (Cossonay, Switzerland. 1919). The first wave in July/August 1918 is not documented because the canton of Vaud did not introduce the compulsory reporting of influenza until September 1918.

Reporting week		Calendar	Newly reported ILI cases		New deaths from ILI
Begin	End	week	n	per 1000	n
July & August			~800		
Introduction of the cantonal reporting obligation for influenza on 24.09.1918					
22.09.18	28.09.18	39	135	11.2	not reported
29.09.18	05.10.18	40	106	8.8	not reported
06.10.18	12.10.18	41	124	10.3	not reported
13.10.18	19.10.18	42	241	20	1
20.10.18	26.10.18	43	209	17.4	3
27.10.18	02.11.18	44	159	13.2	4
03.11.18	09.11.18	45	197	16.4	1
10.11.18	16.11.18	46	103	8.6	0
17.11.18	23.11.18	47	185	15.4	4
24.11.18	30.11.18	48	292	24.3	7
01.12.18	07.12.18	49	287	23.9	6
08.12.18	14.12.18	50	94	7.8	1
15.12.18	21.12.18	51	61	5.1	1
22.12.18	28.12.18	52	73	6.1	3
29.12.18	04.01.19	1	42	3.5	2
05.01.19	11.01.19	2	98	8.2	0
12.01.19	18.01.19	3	66	5.5	0
19.01.19	25.01.19	4	32	2.7	1
26.01.19	01.02.19	5	16	1.3	0
02.02.19	08.02.19	6	17	1.4	0
09.02.19	15.02.19	7	8	0.7	0
16.02.19	22.02.19	8	0	0	0
23.02.19	01.03.19	9	32	2.7	0
02.03.19	08.03.19	10	55	4.6	0
09.03.19	15.03.19	11	7	0.6	1
16.03.19	22.03.19	12	13	1.1	0
23.03.19	29.03.19	13	2	0.2	0
30.03.19	05.04.19	14	2	0.2	0
06.04.19	12.04.19	15	0	0	0
13.04.19	19.04.19	16	0	0	0
20.04.19	26.04.19	17	0	0	0

Bibliography

Autorités de la santé du canton de Vaud (1919) Rapport sur l'épidémie de grippe 1918. Archives Cantonal Vaudoise ARC KVIIIb 27/1 & 27/2. 17, Lausanne

Eidgenössisches Statistisches Amt (1930) Schweizerische Fabrikstatistik vom 22. August 1929. [Eidgenössisches Statistisches Amt], Bern

Eidgenössisches Statistisches Bureau (1919) Die Influenza-Pandemie in der Schweiz 1918/1919. Bull des Schweizerischen Gesundheitsamtes 29:337–344

Imsand M (1972) Câbleries et tréfileries de Cossonay. Câbleries et tréfileries de Cossonay, Cossonay

Koch N (2019) La réaction des autorités vaudoises face à la grippe espagnole

Messerli F-M (1920) L'évolution de l'épidémie de grippe à Lausanne en 1918-1919 : (Rapport historique et statistique). Imprimerie Pache-Varidel & Bron, Lausanne

Schweiz. Statistisches Bureau Eidgenössische Volkszählung vom 1. Dezember 1920. Francke, Bern

Schweiz Schweizerisches Gesundheitsamt Bulletin des Schweizerischen Gesundheitsamtes, 1917-1919. Scheitlin, Bern

Sonderegger C (1991) Die Grippeepidemie 1918/19 in der Schweiz. University of Bern, Bern

Staub K, Jüni P, Urner M, et al (2021) Public Health Interventions, Epidemic Growth, and Regional Variation of the 1918 Influenza Pandemic Outbreak in a Swiss Canton and Its Greater Regions. Ann Intern Med 174:533–539

(1928) Les usines de la S.A. des Câbleries et Tréfileries, à Cossonay-Gare. Bull Tech la Suisse Rom 54:306. https://doi.org/10.5169/seals-41910

(1948) S.A. des Câbleries et Tréfileries Cossonay, 1923-1948. [Verlag nicht ermittelbar], Cossonay
